# The Appetite for Medicine

**Published:** 1868-10

**Authors:** 


THE APPETITE FOR MEDICINE.—It was stated at the Paddington Board of Guardians that 138 gallons of medicine had been dispensed in two months, including 30 gallons of quinine mixture. It was remarked that the inmates were never satisfied unless they were taking medicine.—*London Lancet.*

